# Robot Localization in Water Pipes Using Acoustic Signals and Pose Graph Optimization

**DOI:** 10.3390/s20195584

**Published:** 2020-09-29

**Authors:** Rob Worley, Ke Ma, Gavin Sailor, Michele M. Schirru, Rob Dwyer-Joyce, Joby Boxall, Tony Dodd, Richard Collins, Sean Anderson

**Affiliations:** 1 Department of Automatic Control and Systems Engineering, University of Sheffield, Sheffield S1 3JD, UK; kemasheffield@gmail.com (K.M.); tony.dodd@staffs.ac.uk (T.D.); s.anderson@sheffield.ac.uk (S.A.); 2 Department of Civil and Structural Engineering, University of Sheffield, Sheffield S1 3JD, UK; g.sailor@sheffield.ac.uk (G.S.); j.b.boxall@sheffield.ac.uk (J.B.); r.p.collins@sheffield.ac.uk (R.C.); 3 Department of Mechanical Engineering, University of Sheffield, Sheffield S1 3JD, UK; Michele.Schirru@ac2t.at (M.M.S.); r.dwyer-joyce@sheffield.ac.uk (R.D.-J.)

**Keywords:** robot localization and mapping, SLAM, pose-graph optimization, pipe inspection robot

## Abstract

One of the most fundamental tasks for robots inspecting water distribution pipes is localization, which allows for autonomous navigation, for faults to be communicated, and for interventions to be instigated. Pose-graph optimization using spatially varying information is used to enable localization within a feature-sparse length of pipe. We present a novel method for improving estimation of a robot’s trajectory using the measured acoustic field, which is applicable to other measurements such as magnetic field sensing. Experimental results show that the use of acoustic information in pose-graph optimization reduces errors by 39% compared to the use of typical pose-graph optimization using landmark features only. High location accuracy is essential to efficiently and effectively target investment to maximise the use of our aging pipe infrastructure.

## 1. Introduction

**Figure 1:**
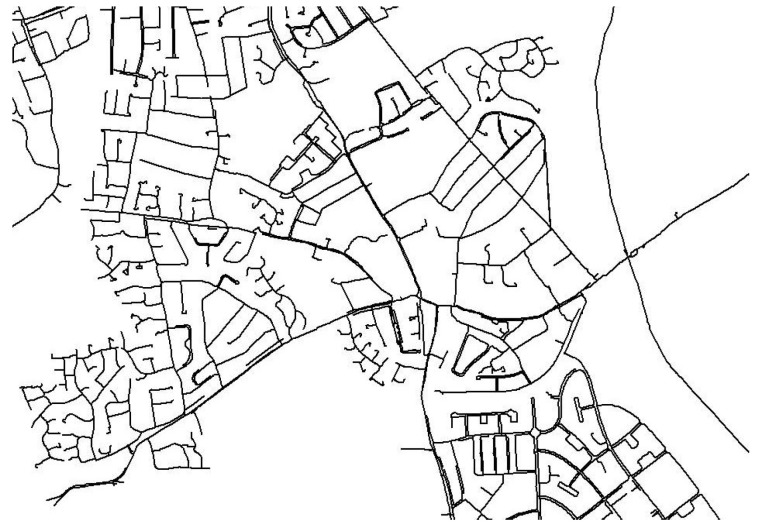
An example water supply pipe map for a region in the UK. The range of pipe layouts can be seen: straight pipes, curved pipes, and pipes with sharp corners. The scale of the map is 2 km from the bottom to the top.

Water distribution and wastewater infrastructures are aging and are in regular need of costly maintenance. The buried pipe network for water and wastewater in the UK is around 800,000 km in combined length [1], and the UK’s water infrastructure is the product of over 250 billion pounds of investment [2]. It is estimated that over 3000 million litres of water are lost to leaks every day in the UK [3]. Mobile robots could be used for autonomous, persistent monitoring of a buried pipe network, locating faults and returning to an operator to report the information. The aim would be to reduce the resources required for manual inspection, to improve the spatial coverage and frequency of inspection, and thereby to improve the overall resilience of the water services. An example water pipe network that such a robot would operate in is given in Figure 1.

A principal challenge for such a robotic system would be to reliably and precisely estimate the location of a fault in a buried pipe in the absence of long-range communication, a global positioning system, and a magnetic compass in a pipe network that is relatively featureless. This is a simultaneous localization and mapping (SLAM) problem [4,5], where the pose (and sometimes the previous trajectory) of the robot and the location of a fault need to be estimated. The robotic system must operate in pipes with a typical diameter of 150 mm or less and must be smaller than this diameter to not affect the flow of water. The scale of the robotic system therefore limits the design to high-noise sensors, uncertain motion, and low computational power. However there are advantages in that the buried pipe environment is simple, constrained, largely unchanging with time, and somewhat mapped as shown in Figure 1, giving prior knowledge with some uncertainty.

Conventional sensors, such as cameras, sonars, lasers, ultrasound range-finders, and inertial measurement units (IMUs), have been applied to the problem of localization in pipes in a number of ways. Visual odometry has been performed using images from a camera focused on the pipe wall [6] or along the axis of the pipe [7]. Detection and recognition of junctions and corners has also been developed [8] and augmented by structured light [9,10], use of time-of-flight imagery [11], and projection of a laser spot array [12] or projected radial lines [13].

Although these visual methods are effective, there are difficulties in adapting the required hardware to the constraints of the pipe environment. For example, a light source is required, which consumes additional power, and the camera might become obscured in the dirty environment of sewers. This motivates exploration of alternative sensing technologies for pipe environments.

Dead reckoning methods based on inertial measurement units (IMUs) and odometry have been used with drift correction to estimate location in pipe networks. IMUs typically use a magnetometer and accelerometer to give absolute measurements of heading and downwards direction and use gyroscopes for rate of change in angles. In the pipe environment, it is expected that the magnetometer component will not give a reliable measurement of heading angle, and so, the measurement using the gyroscope alone will suffer from drift due to integrated error [14,15]. Therefore, methods have been developed to use IMU measurements in combination with tethers measuring distance travelled [16,17], knowledge of known landmark positions [18,19], and knowledge of the length of pipe sections [20] in order to correct drift. IMUs have also been used to detect features such as junctions and corners [15,21,22].

To compensate for the otherwise feature-sparse nature of the pipe environment, alternative sensing methods can be used. These methods include the use of a tether cable to measure distance travelled [17] and, similarly, using latency in the arrival of a sound wave from a fixed source [23]. However, these methods require a tether to the robot, which limits the desired autonomy in this application. Another approach is to create a spatial field using an emitter at a fixed point using radio frequency waves [24]. While this method has been shown to achieve accurate localization in the intended application in large pipes, the requirement for the emitter to be located in a fixed position separate from the receiver on the mobile robot again limits the desired autonomy. The reduction in autonomy given by these alternative sensing methods motivates the use of features in the environment that can be detected using only one non-tethered robot.

In previous work, we have used a hydrophone emitter-receiver positioned on a robot to perform localization [25,26]. The hydrophone emits a sound wave that interacts with a metal pipe in a way that varies over space and so can be used to recognise location. The limitation of previous work is that only *online* localization is done, which only estimates the current location. Instead, in this work, the full trajectory is estimated, which would allow better estimation of the location of faults in the pipe detected by the robot. The objective information available and methods used here are therefore different. We present a novel solution here for full trajectory estimation using a pose-graph optimization method [27,28,29,30,31].

Pose-graph optimization uses efficient sparse nonlinear least squares methods to estimate the robot location. These back-end estimation algorithms tend to be commonly usable across application domains. However, the front-end construction of the pose graph from sensor data tends to be unique to the application, which is the case here. We propose and evaluate four alternative methods for constructing the pose graph from the spatially varying hydrophone acoustic signal: 1. linear fit, 2. quadratic fit, 3. cross correlation and kernel cross correlation [32], and 4. phase correlation.

Similar pose-graph optimisation methods have been developed for use with an ambient distorted magnetic field in an application in drilling robots [14] and ambient electric and magnetic fields in a two-dimensional indoor environment [32,33,34,35]. The contribution of this paper is the presentation of an approach with two key differences to these pieces of work. Firstly, the focus in this work is on achieving a soft loop-closing effect rather than only explicitly matching subsets of measurements to find loop-closures. This exploits the continuous nature of the measured spatial field to avoid the requirement for robustness in feature detection and matching. Secondly, where correlation approaches are used in a similar way to the related work to estimate loop-closures, further development has been made to estimate uncertainty in the loop-closure estimates.

In order to evaluate the pose-graph optimization algorithm, we use data recorded in a 5-m long metal pipe filled with water, which is an order of magnitude longer than in our previous work [25,26]. We also analyse the performance of the algorithm in simulations over much larger scales than we could do in a laboratory, which relates more closely to real-world pipe networks. The simulations are driven by synthetic data that are derived from experimental data to provide realistic evaluations.

The paper is structured as follows. The acoustic spatial field, experimental data, and synthetic data are described in Section 2. In Section 3, the pose-graph optimization problem is defined and the estimation algorithm is derived. This includes the methods of adding spatial field information to the pose graph. The results from experimental work and simulations that demonstrate the effectiveness of the proposed pose-graph optimization algorithm are given in Section 4. A discussion of the work is given in Section 5. Finally, conclusions are given in Section 6.

## 2. Methods: Acoustic Signal for Robot Localisation

### 2.1. One-Dimensional Acoustic Signal

Metal pipes exhibit vibrations when excited by a sound wave produced by a hydrophone in the kilohertz range. This vibration tends to vary over space and so can be used like a map in loop closing. We define the spatial field used in localization in this paper as a one-dimensional acoustic signal varying along the pipe, observed at sample time *t*:

(1) $$s_{t}=\frac{1}{n_{\omega }}\sum_{k=\omega_{l}}^{\omega_{u}}a_{k}$$

which is the average pipe vibration amplitude over a set of $n_{\omega }$ frequencies, in the range $[\omega_{l}$, $\omega_{u}]$, where the vibration amplitude spectrum is defined as

(2) $$A=\left[a_{0},a_{1},\ldots ,a_{N/2}\right]=\frac{1}{N}\left[|M_{0}\left|,2\right|M_{1}\left|,\ldots ,2\right|M_{N/2-1}\left|,\right|M_{N/2}|\right]$$

where

(3) $$M_{k}=\sum_{n=0}^{N-1}m_{n}e^{-\frac{j2\pi }{N}kn}$$

is the discrete Fourier transform (DFT) of $m_{k}$, which is the received signal from the hydrophone, where *k* denotes sample index and *N* is the number of samples.

We make the assumption that the acoustic signal $s_{t}$ can be treated as a measurement at sample time *t*. This is justified because the sensing is very fast compared to the robot motion and robot localization, as the acoustics operate in the tens of kilohertz range. This is similar to a lidar scan in mobile robots being treated as a single set of measurements at sample time *t*.

### 2.2. Experimental Data

Experimental data have been recorded using a hydrophone unit to measure the spatially varying acoustic signal $s_{t}$ in a 5-m-long water-filled pipe. The sensor unit consists of a pair of hydrophones (Bruel & Kjaer Miniature Hydrophone Type 8103). A waveform at around 3.7 MHz is amplified and input to the first hydrophone. The second hydrophone measures the vibration of the metal pipe, and this signal is amplified and logged. This process is repeated a number of times as the pair of hydrophones are moved along the length of the 5-m pipe. The sensor platform is precisely moved in intervals of 1 centimetre using a pulley system controlled by a stepper motor, rather than using sensors mounted to a robot. This creates a ground truth for robot motion and can be used to create noise-corrupted synthetic data, as described in Section 2.3.

**Figure 2:**
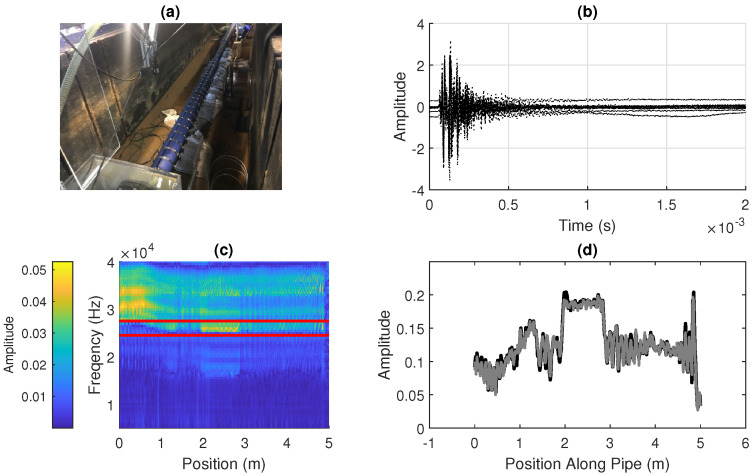
The experimental measurement of a spatially varying acoustic property along the length of a water pipe: (**a**) a photograph of the experiment at the University of Sheffield; (**b**) vibration time-response from points along a pipe, recorded using a hydrophone to excite the vibration and to record the response amplitude; (**c**) frequency spectra for a specific band (5 kHz to 40 kHz) for each position along a pipe, with the amplitude within a window (in this case 24.5 kHz to 27.5 kHz, shown by the red lines) summed to give a spatially varying signal along the pipe; and (**d**) the resulting spatially varying signal along the length of the pipe, where two sets of measurements are shown superimposed and it can be seen that the signal is largely the same in each case.

The data in this work are similar in nature to that reported in our previous work [25,26]; however, it has been extended from a 0.4-m pipe to a 5-m pipe, shown in Figure 2a. The process of acquiring the spatially varying signal is the same as in a previous work [25]. Examples of the time-domain acoustic response recorded at different points along the pipe are shown in Figure 2b. The Fourier transforms of these responses are found, which are shown in Figure 2c.

Summing the amplitude of the responses in a particular frequency band, as described in Equations (1)–(3), gives a scalar quantity which varies along the pipe, which can be seen in Figure 2d. In this work, the frequency band chosen is 24.5 kHz to 27.5 kHz in this work, as illustrated in Figure 2c; however, in practice, this may need to be varied depending on the properties of the pipe, fluid, and surrounding material. A further front-end process may be needed to assess the full spectrum to select a frequency band which gives sufficient variation in amplitude. While this process has not been investigated in this work, it is not expected to affect the methods presented here as the spatially varying signal is used as an arbitrary quantity for the purposes of localization. This resulting spatially varying signal is largely consistent over time, as shown by the comparison of two sets of measurements in Figure 2d.

### 2.3. Synthetic Data

To evaluate the robot localisation method over additional data sets and a larger spatial scale, synthetic data sets were created using the experimental data.

**Figure 3:**
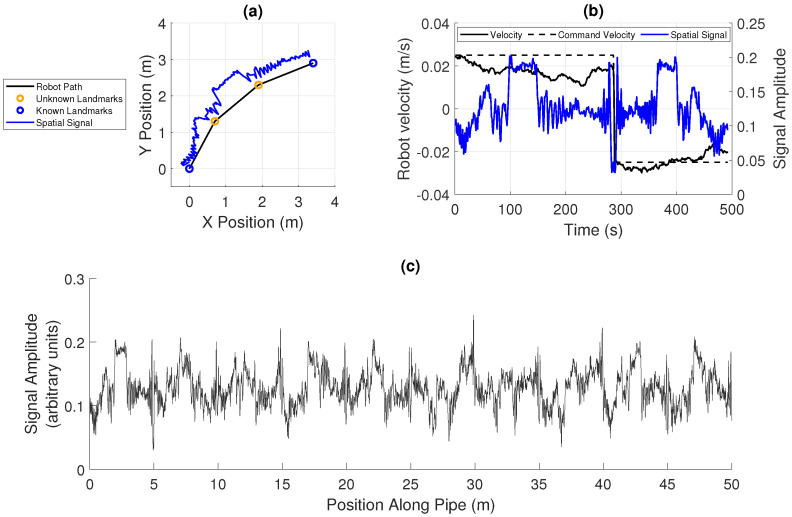
The creation of synthetic 1D data based on the experimental data: (**a**) the spatially varying acoustic signal superimposed on the trajectory of the robot along a pipe, illustrating how the signal would vary in the semi-two-dimensional space; (**b**) the motion of the robot along the pipe in each direction, where a fixed command velocity is given, the robot’s actual velocity drifts with additive noise, and the spatial signal can be seen to be warped due to this drift; and (**c**) a larger set of spatial signal data, simulated based on the experimental data.

Figure 2d shows the experimentally measured spatial signal, and Figure 3a shows this spatial signal mapped into a simulated two-dimensional map. In this case, where there are corners in the pipe, a measurement of the heading angle of the pipe using a gyroscope in an IMU could be used to detect features. Landmarks connected to the surface such as manholes may have known positions, while the position of corners or junctions may not be known accurately.

The robot’s motion is modelled as discrete time steps along the pipe, considering only the motion along the axis of the pipe, and a measurement of the spatial field is taken at each point. A trajectory is therefore made up of a set of discrete positions $x_{0:T}^{r}$ and corresponding measurements of the spatial signal $s_{0:T}$ and landmarks $z_{0:T}$. The robot’s velocity between each step is modelled as a random process which results in drift in velocity from integrated additive Gaussian noise, giving uncertainty in the position illustrated in Figure 3b, similar to that in another work [23]. This is intended to model the effect of variation in the robot’s pipe axis velocity depending on the small robot’s real orientation and forward velocity within the pipe.

Figure 3c shows a signal simulated using the experimental data in Figure 2d, which is used to extend assessment of the developed methods to a challenging signal with some periodicity and similarity between sections.

## 3. Methods: Robot Localisation Using Pose Graph Optimization

### 3.1. Conventional Pose Graph Optimization for Pipe Robots

In this section, we define the conventional pose-graph optimization problem that can be used to localise a robot in a pipe with respect to conventional features, such as pipe junctions and corners.

We assume that a robot moves along a pipe according to the motion model:

(4) $$x_{t}^{r}=g\left(u_{t},x_{t-1}^{r}\right)+w_{t}$$

where $x_{t}^{r}$ is the robot position in one dimension along the axis of the pipe at sample time *t*, $u_{t}$ is an input, *g* is the nonlinear state transition function, and $w_{t}$ is Gaussian random state noise where $w_{t}∼N\left(0,R_{t}\right)$.

The robot is able to detect landmarks such as junctions and corners in the pipe section, where we assume data association is known, giving the following model:

(5) $$z_{t}^{i}=h\left(x_{t}^{r},x^{i}\right)+v_{t}$$

where $z_{t}^{i}$ is the measurement at time *t* of landmark feature *i*, $x^{i}$ refers to environment features *i*, *h* is the nonlinear measurement function, and $v_{t}$ is Gaussian random measurement noise where $v_{t}∼N\left(0,Q_{t}\right)$. In this case, the robot only makes measurements of landmarks when they are nearby, so for many time indices *t*, there will be no measurements.

The typical pose-graph optimisation problem [36] is defined by the cost function:

(6) $$\begin{array}{cc} J & =x_{0}^{rT}\Omega_{0}x_{0}\\ & +\sum_{t}^{T}{\left(x_{t}^{r}-g\left(u_{t},x_{t-1}^{r}\right)\right)}^{T}R_{t}^{-1}\left(x_{t}^{r}-g\left(u_{t},x_{t-1}^{r}\right)\right)\\ & +\sum_{t}^{T}\sum_{i}^{I}{\left(z_{t}^{i}-h\left(x_{t}^{r},x^{i}\right)\right)}^{T}Q_{t}^{-1}\left(z_{t}^{i}-h\left(x_{t}^{r},x^{i}\right)\right) \end{array}$$

where $x_{0}$ is the initial state which has uncertainty $\Omega_{0}^{-1}$ and where *T* and *I* are the number of time steps and features, respectively. The solution is sought as

(7) $$x^{*}=\underset{x}{arg min}J\left(x\right)$$

where x is given by

(8) $$x=\left[\begin{array}{c} x_{0:T}^{r}\\ x^{0:I} \end{array}\right]$$

This optimization problem is solved iteratively. The solution can either use analytic methods, where the gradient (or Jacobian) in the solution space is known, or can use numerical methods to compute this gradient.

### 3.2. Pose Graph Optimization Using an Acoustic Signal

It is desired to simultaneously optimize the trajectory estimate with respect to the feature measurements and acoustic signal measurements. This would allow the incorporation of prior knowledge, more feature measurements, or more signal measurements without further alteration of the problem.

Therefore, to incorporate the acoustic signal term $s_{t}$ into the pose-graph optimisation problem, we augment the cost function with an additional term ϕ which defines a measure of inconsistency in the estimated spatial signal measurements along the pipe. This is given by

(9) $$\begin{array}{cc} J & =x_{0}^{rT}\Omega_{0}x_{0}^{r}\\ & +\sum_{t}^{T}{\left(x_{t}^{r}-g\left(u_{t},x_{t-1}^{r}\right)\right)}^{T}R_{t}^{-1}\left(x_{t}^{r}-g\left(u_{t},x_{t-1}^{r}\right)\right)\\ & +\sum_{t}^{T}\sum_{i}^{I}{\left(z_{t}^{i}-h\left(x_{t}^{r},x^{i}\right)\right)}^{T}Q_{t}^{-1}\left(z_{t}^{i}-h\left(x_{t}^{r},x^{i}\right)\right)\\ & +\sum_{t}^{T}\phi {\left(t,x_{0:T}^{r},s_{0:T}\right)}^{T}P_{t}^{-1}\phi \left(t,x_{0:T}^{r},s_{0:T}\right) \end{array}$$

where $x_{0:T}$ and $s_{0:T}$ are positions and acoustic measurements along the pipe, and $P_{t}$ is the covariance of this field measurement model noise. The remaining terms are the same as in Equation (6), and again, the cost function is minimize as in Equation (7).

The function ϕ generally has the following form:

(10) $$\phi \left(t,x_{0:T}^{r},s_{0:T}\right)=y\left(t,x_{0:T}^{r},s_{0:T}\right)-f\left(t,x_{0:T}^{r},s_{0:T}\right)$$

where *y* is a function giving some measurement of the signal s and *f* is a function giving the expected value of that measurement, much like the terms *z* and *h*, respectively.

In the specific implementations of this function presented in the following section, $y(t,x_{0:T}^{r},s_{0:T})$ is either the signal value $s_{t}$ or a measured distance equal to zero between two matching points, and $f(t,x_{0:T}^{r},s_{0:T})$ is either the value of a local best fit function over a region of *x* or the distance between two matching points in the current estimate of x.

Unlike the typical cost function terms, this term does not necessarily give a measure of difference in distance and can instead be a measure of distance in the abstract spatial signal quantity. Also, unlike the typical measurement term *z*, the measurement component of this term, *y*, is dynamic and is updated at each iteration of the optimization.

**Figure 4:**
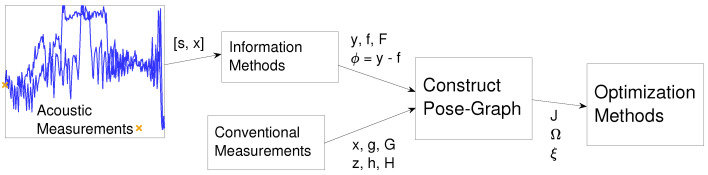
An illustrative block diagram outlining the process described in Section 3: the symbols on each arrow describe the variables output from the functions described by a block. Each symbol is defined within Section 3. A typical pose-graph optimization would optimize a pose graph constructed using conventional measurements. This work augments the pose graph with information from acoustic measurements. The iterative nature of the process is not illustrated; the whole process is repeated for each new estimated trajectory *x* output from the optimization.

The process described here and in the rest of Section 3 is illustrated in Figure 4.

### 3.3. Spatial Signal Information Methods

This section presents a number of specific implementations of the general cost function term in Equation (10). In this application, the robot moves twice along a pipe, recording measurements of the spatial signal of vibration amplitude. These measurements of the spatial signal can be used to improve estimatation of the robot’s trajectory.

Assuming the robot has travelled along the pipe twice, the signal can be split into two sets: one for motion in each traversal of the pipe. This method could be extended simply to signals from multiple sets if the robot has moved along the pipe multiple times. Perfect knowledge of the robot’s trajectory would mean that the two signals would be aligned and would correspond to the true spatial signal. However, the signals are warped along the spatial axis due to uncertainty in the position along the pipe at which each measurement was taken. Essentially, aligning the two signals within the other constraints of the pose graph increases the likelihood of accuracy of the corresponding set of poses and is equivalent to finding and matching features as done in typical robot localization.

In this section, methods for incorporating information from the spatial signal measurements to the pose graph are described, represented by the *information methods* block in Figure 4. Throughout the section, $x_{t}$ refers to the current position estimate of the pose at time *t* and $s_{t}$ refers to the measurement of the spatial signal at time *t*. $f(t,x_{0:T}^{r},s_{0:T})$ and $y(t,x_{0:T}^{r},s_{0:T})$ (from Equation (10)) are referred to as $f_{t}$ and $y_{t}$, respectively, and are defined in each of the following subsections for each information method.

#### 3.3.1. Two-Point Linear Fit Prediction

**Figure 5:**
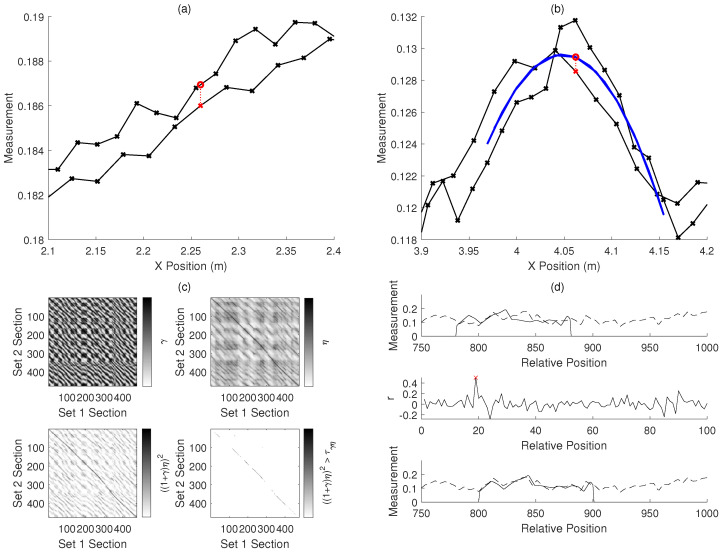
Illustrations of methods of taking information from the measurements of the spatially varying acoustic property: the information is used in the pose-graph optimization to improve the robot trajectory estimate. (**a**) The function of the *linear fit* method: the two sets of poses and measurements (black crosses) should be aligned. The measurement at the pose in red (at 2.260 m) is predicted to be at the value of the red circle on the line between the two neighbouring points (at 2.255 m and 2.276 m). (**b**) The function of the *quadratic fit* method: the two sets of poses and measurements (black crosses) should be aligned. The measurement at the pose in red (at 4.062 m) is predicted to be at the value of the red circle on the quadratic fit of the nearby points (shown in blue). (**c**) An illustration of the cross-correlation method: an example of the matching parameters used to find correspondences in the two sets of data split into the labelled sections. γ is the cross-correlation coefficient between the sections, η is the inverse of the sum of squared error between the sections, and ${\left(\left(1+\gamma \right)\eta \right)}^{2}$ is the parameter used to determine matches between sections by comparison to a threshold $\tau_{\gamma \eta }$. (**d**) An illustration of the phase-correlation method used to find the offset between the sections of the signals in two sets of measurements: the two misaligned sections ($s_{1}$ and $s_{2}$) are shown in the first plot. The second plot shows the cross-power function *r*, where the position of the largest value should correspond to the position shift which best aligns the two sections. The aligned sections are shown in the third plot.

For each pose $x_{t}^{r}$, the nearest two poses in the current estimate, $x_{a}$ and $x_{b}$, and corresponding signals, $s_{a}$ and $s_{b}$, are found. It is predicted that $y_{t}=s_{t}$ will lie on the straight line drawn between the points *a* and *b* as given by

(11) $$f_{t}={\hat{s}}_{t}=\left(x_{t}^{r}-x_{a}\right)\frac{\left(s_{b}-s_{a}\right)}{\left(x_{b}-x_{a}\right)}+s_{a}$$

and illustrated in Figure 5a. For incorporation with the analytical optimization method, the Jacobian of $f(t,x_{0:T}^{r},s_{0:T})$ is needed with respect to $x_{0:T}^{r}$. This is given by

(12) $$\begin{array}{cc} \; & F_{t}=\left[\begin{array}{c} \frac{\partial f_{t}}{\partial x_{t}^{r}},\frac{\partial f_{t}}{\partial x_{a}},\frac{\partial f_{t}}{\partial x_{b}} \end{array}\right]\\ & =\left[\begin{array}{c} \frac{\left(s_{b}-s_{a}\right)}{\left(x_{b}-x_{a}\right)},\frac{\left(x_{b}+x_{t}^{r}\right)\left(s_{b}-s_{a}\right)}{{\left(x_{b}-x_{a}\right)}^{2}},\frac{\left(x_{a}-x_{t}^{r}\right)\left(s_{b}-s_{a}\right)}{{\left(x_{b}-x_{a}\right)}^{2}} \end{array}\right] \end{array}$$

which excludes the matrix that would be used to map each term in the Jacobian to the correct position in the vector of poses. For each iteration, this prediction is made for each pose and the resulting information is added to the pose graph. In principle, a prediction of the position of the pose $x_{t}^{r}$ can be made directly from the linear fit. However, in practice, for two signals of similar value, the gradient of the linear fit is very small and the prediction can be very far from the current estimate, leading to instabilities in the optimization.

#### 3.3.2. Quadratic Fit Prediction

For each pose, the poses and measurements within a window distance $w_{t}$ are used to create a local quadratic fit, parameterized by $p_{2}$, $p_{1}$, and $p_{0}$. It is predicted that $y_{t}=s_{t}$ will lie on this quadratic curve, as given by

(13) $$f_{t}={\hat{s}}_{t}=p_{2}x_{t}^{r2}+p_{1}x_{t}^{r}+p_{0}$$

and illustrated in Figure 5b. A multi-scale quadratic fit can be done, where the prediction is made for a number of window sizes and all the resulting information is incorporated into the optimization.

The Jacobian is needed to incorporate the prediction into the analytical optimization; however, in this case, the calculation is difficult as the quadratic parameters $p_{2}$, $p_{1}$, and $p_{0}$ are all functions of $x_{t}^{r}$. Instead, $p_{2}$ and $p_{1}$ are treated as constants and the Jacobian can be computed as

(14) $$F_{t}=2p_{2}x_{t}^{r}+p_{1}$$

This approximation of $p_{2}$, $p_{1}$, and $p_{0}$ as constants means that the cost associated with this prediction is only applied to one pose: $x_{t}^{r}$. There are therefore limitations on the optimization methods that can be used (which are described in Section 3.4) as the information cannot be incorporated into Matlab’s *PoseGraph Optimization* functions. The approximation also introduces error in the analytical optimization; however, the method qualitatively appears to work effectively.

As in the linear fit method, in principle, a prediction of the position of $x_{t}^{r}$ could be made directly from the quadratic fit. However, as well as the problems in the linear fit case, signals outside of the measurement axis range of the quadratic function would have an undefined position estimate, so it is not possible to use in many cases.

#### 3.3.3. Cross-Correlation Matching

The signal is split into two sets ($\left[x_{p1}^{r},s_{p1}\right]$, $\left[x_{p2}^{r},s_{p2}\right]$), one for each traversal of the pipe, and matches between corresponding poses in each set are found using cross-correlation, as done in a similar work [14].

Interpolation is used to create two sets of points ($\left[x_{q1}^{r},s_{q1}\right]$, $\left[x_{q2}^{r},s_{q2}\right]$) of equal number $N_{q}$. The normalized cross-correlation coefficient is then found between subsets of points ($\left[x_{1}^{r},s_{1}\right]$, $\left[x_{2}^{r},s_{2}\right]$) of smaller number ($N_{s}$ and $N_{y}$), which are taken across the whole set of points. The normalized cross-correlation coefficient is given by

(15) $$\gamma =\frac{\sum_{x}\left(s_{1}\left(x\right)-\bar{s_{1}}\right)\left(s_{2}\left(x\right)-\bar{s_{2}}\right)}{\sqrt{\sum_{x}{\left(s_{1}\left(x\right)-\bar{s_{1}}\right)}^{2}\sum_{x}{\left(s_{2}\left(x\right)-\bar{s_{2}}\right)}^{2}}}$$

and is a measure of the match between sets of points. Alternatively, as the magnitude of the signals is expected to be consistent at each point along the pipe, the sum of difference between the two subsets can also be used as a measure of a match, described by

(16) $$\eta ={\left[\sum_{x}{\left(s_{1}\left(x\right)-s_{2}\left(x\right)\right)}^{2}\right]}^{-1}$$

The results of Equations (15) and (16) are shown in Figure 5c. A value ϵ is found from

(17) $$\epsilon ={\left(\left(1+\gamma \right)\eta \right)}^{2}$$

If ϵ is greater than a threshold $\tau_{\gamma \eta }$, the poses corresponding to $s_{1}$ and $s_{2}$ are considered matches and are added to the cost function term in Equation (10) at poses corresponding to the centre of the matching sections, ${\tilde{x}}_{1}^{r}$ and ${\tilde{x}}_{2}^{r}$. The measured distance and expected distance are given by

(18) $$y_{t}=0$$

(19) $$f_{t}={\tilde{x}}_{1}^{r}-{\tilde{x}}_{2}^{r}$$

Note that the choice to match the poses at the centre of each sequence is unlikely to be accurate; however, further matching within these sequences would give an increase in complexity.

The correlation process to estimate the matching points ${\tilde{x}}_{1}^{r}$ and ${\tilde{x}}_{2}^{r}$ is dependent on a number of poses in $x_{0:T}^{r}$; however, the simple approximation of $f_{t}$ in Equation (19) allows for easy computation of the Jacobian as

(20) $$F_{t}=\left[\begin{array}{c} 1,-1 \end{array}\right]$$

The covariance $P_{t}$ can be set to be inversely proportional to the product of γ and η, so that stronger matches are effectively weighted higher in the cost function in Equation (6).

#### 3.3.4. Kernel Cross-Correlation Matching

An improvement to linear cross-correlation described in the previous section may be to use *kernel cross-correlation* [37], which should be more robust to signal noise and distortion. This is implemented as follows, assuming a similar process as described in the previous section is used to create a number of subsets of points given by ($\left[x_{1}^{r},s_{1}\right]$, $\left[x_{2}^{r},s_{2}\right]$).

A kernel vector is defined as $\kappa_{s_{i}}\left(s_{j}\right)$ between two sets of points, using a Gaussian kernel with a parameter σ. $\kappa_{s_{1}}\left(s_{1}\right)$ is computed, and a correlator $B^{*}$ is defined in the Fourier domain using

(21) $$B^{*}=\frac{A}{K_{s_{1}}\left(s_{1}\right)+\lambda }$$

where $K_{s_{1}}\left(s_{1}\right)$ is the Fourier domain kernel vector and A is the target response in the Fourier domain, where the corresponding spatial domain response α is defined as a vector where all elements are equal to 1, indicating a match.

For each test subset $s_{2}$ and corresponding Fourier domain kernel vector $K_{s_{1}}\left(s_{2}\right)$, a similarity measure is found using

(22) $$\zeta =\max F^{-1}\left(K_{s_{1}}\left(s_{2}\right)\circ B^{*}\right)$$

where ∘ denotes the element-wise multiplication of the two vectors. In this work, the value $\sqrt{\zeta }$ is used in the same was as the value ϵ as described in the previous section, and the same process is used to add information to the pose graph.

#### 3.3.5. Phase-Correlation Matching

As in the cross-correlation method, the signal is split into two sets and interpolation is used to find equivalent sets with an equal number of points. Phase-correlation is used to find the offset, if it exists, between smaller subsets of points. The phase-correlation method is described by

(23) $$C=\frac{S_{1}\circ S_{2}^{*}}{|S_{1}\circ S_{2}^{*}|}$$

(24) $$c=F^{-1}\left[C\right]$$

(25) $$\Delta x=\underset{x}{arg max}c\left(x\right)$$

where $S_{1}$ and $S_{2}$ are the Fourier transforms of $s_{1}$ and $s_{2}$, $S_{2}^{*}$ is the complex conjugate of $S_{2}$, $F^{-1}$ is the inverse Fourier transform, and ∘ denotes the element-wise multiplication of the two vectors. This is illustrated in Figure 5d.

Again, as in the cross-correlation method, the difference between the two sequences $s_{1}$ and $s_{2}$ is used, as given in Equation (16). As a large value of the peak, $c_{max}$, in the cross-power function c corresponds to a good match between sequences, if the product of η from Equation (16) and $c_{max}$ is larger than a threshold $\tau_{r\eta }$, the match is added to the cost function term in Equation (10) between poses corresponding to the centre of the compared sections, offset by Δx. The measured and predicted values used in Equation (10) are given by

(26) $$y_{t}=\Delta x$$

(27) $$f_{t}={\tilde{x}}_{1}^{r}-{\tilde{x}}_{2}^{r}$$

Similarly to the cross-correlation method, the covariance can be set to be inversely proportional to the product of $c_{max}$ and η, and Equation (20) can be used as the Jacobian in this method too. The same assumptions regarding the choice to match the two poses ${\tilde{x}}_{1}^{r}$ and ${\tilde{x}}_{2}^{r}$ are also made in this case.

### 3.4. Optimization Solution Methods

To minimise the cost function in Equation (9), the original GraphSLAM algorithm [36] uses the information form described by the parameters Ω and ξ, which is an inverse to the covariance form, to parameterize the probabilistic estimation. This relationship is respectively defined as $\Omega =\Sigma^{-1}$ and ξ=Ωμ for the covariance, Σ, and the mean, μ, of the probability distribution of the pose estimate $x_{0:T}^{r}$.

The terms *g*, *h*, and *f* in the quadratic cost function in Equation (9) can be linearized to derive an equation which is quadratic in *x*, the variable which is to be estimated. This gives the linearised cost function:

(28) $$J_{l}=k+x_{0:T}^{rT}\Omega x_{0:T}^{r}+x_{0:T}^{rT}\xi$$

where Ω is the information form matrix, which is a function of the uncertainty in measurements and the Jacobian of the expected measurement models, and ξ is the information form vector, which is a function of the same variables and the measurements, expected measurements, and current pose estimate.

The construction of this cost function corresponds to the *construct pose-graph* block in Figure 4. The cost function in Equation (28) can be minimized by using the relation between the information form and covariance form to iteratively update the estimate of $x_{0:T}^{r}$. This optimization corresponds to the final block in Figure 4.

The use of analytical optimization requires derivation of an analytical Jacobian for the measurement and motion models. These tend to be known for the case of typical mobile robot models; however, the addition of the spatial field to the estimation can require calculations which are difficult to derive in the case where large numbers of poses are used and can be prone to instability. Therefore, simplifications have to be made when using this optimization method with some of the information methods described in Section 3.3.

The Gauss–Newton method, Levenbert–Marquardt method, and trust region methods, as examples, can be used to minimize a sum of squares function [38] by numerically computing the Jacobian rather than needing it to be explicitly defined. These methods as well as others are implemented in Matlab’s *Optimization Toolbox* in the lsqnonlin function and Matlab’s *Navigation Toolbox* in the optimizePoseGraph function.

## 4. Results

The novel pose-graph optimization algorithm using an acoustic signal defined in Equation (9) is evaluated in this section, comparing the four methods proposed above for incorporating the acoustic signal into pose-graph optimization: *linear fit*, *quadratic fit*, *cross-correlation*, and *phase-correlation*. These are compared to pose-graph optimization without using an acoustic signal, just using landmark *features* at each end of the pipe, as defined in Equation (6). These pose-graph optimization methods are also compared to *dead reckoning*.

The experimental data (shown in Figure 2d) and synthetic data (which is derived from the experimental data, as described in Section 2 and shown in Figure 3c), is used to compare the effectiveness of the developed localization methods. The objective is to estimate the location of a robot that has travelled twice along a pipe, once in each direction.

The uncertainty in the robot’s position is modelled by integrated random normally distributed noise on the robot’s velocity, which results in a drifting velocity, as shown in Figure 3b. The variance of the normally distributed noise added to the motion at each time step is equal the noise magnitude multiplied by the command motion. The robot’s motion is constrained between 0.2 and 1.8 times the command motion. The magnitude of the noise is set to be proportional to the length of the pipe, so the difference between each set of data is only the shape of the spatial signal, as it is the performance of the methods for different shapes of data that is to be compared.

Two metrics, the variance in error and the error, are used to compare results between different methods. The variance in estimation at each point along the pipe is a measure of how consistent the estimate is and is effectively what the information methods aim to reduce directly. The error in the estimate is a measure of the accuracy of the estimate and is desired to be reduced overall; however, there is no means to do this directly. Some quantitative results are presented here, followed by a discussion in the following section.

### 4.1. Comparison of Methods

**Figure 6:**
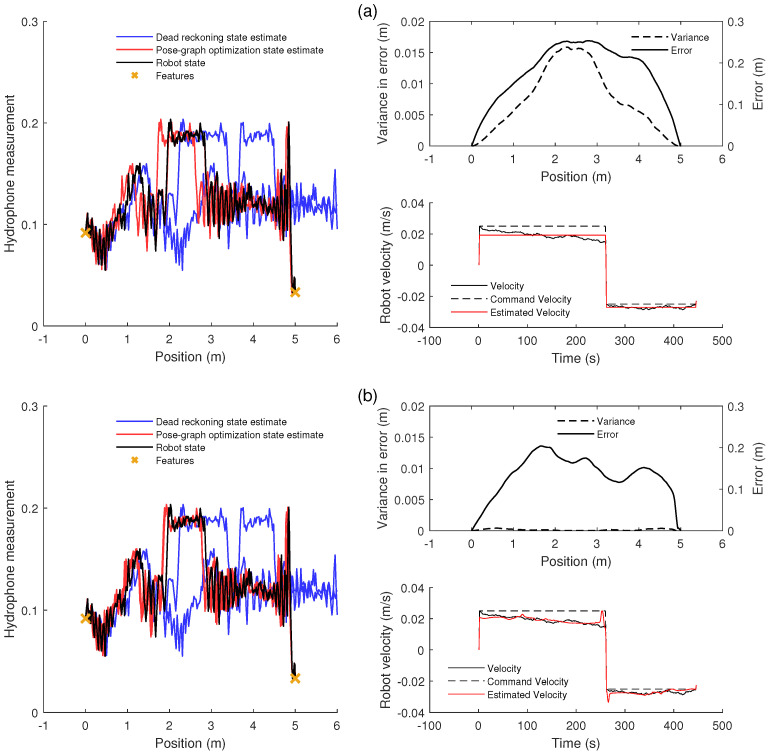
Results from using the developed methods to improve the trajectory estimate in a single 1D pipe: (**a**) an example of the results of the one-dimensional pipe trajectory optimization, using typical pose-graph optimization, illustrated by the resulting spatial signal estimation. The non-optimized estimate (blue) shows large inconsistency and error. The optimized estimate (red) shows some consistency, as the two signals are within the boundaries of the pipe, and accuracy, as the signals coincide well with the true spatial signal (black). The variance in the error, a measure of the inconsistency of the estimates, is shown along with the magnitude of the error for each point along the pipe. The velocity estimate is also shown, showing the drifting velocity in black and the simple estimate of a fixed velocity in red. (**b**) An example of the results of the one-dimensional pipe trajectory optimization illustrated by the resulting spatial signal estimation using the quadratic fit prediction and phase-correlation methods: The non-optimized estimate (blue) shows large inconsistency and error. The optimized estimate (red) shows consistency, as the two signals are aligned, and accuracy, as the signals coincide well with the true spatial signal (black). The variance in error, a measure of the inconsistency of the estimates, is shown along with the magnitude of the error for each point along the pipe. The velocity estimate is also shown, showing the estimated velocity in red closely matching the drifting velocity in black.

An example of the use of the optimization methods is shown in Figure 6. The optimized trajectory shown in Figure 6a uses only the features at the ends of the pipe to improve the estimate. The original *dead reckoning* estimate can be seen to differ substantially from the true trajectory; the estimated trajectory is outside of the length of the pipe, and the shape of the acoustic signal is shifted along the pipe. The original estimate can also been seen as inconsistent as the shapes of the signal recorded in each direction are not aligned. The *pose-graph optimization* trajectory using only the *features* at the end of the pipe can be seen to be much more accurate and consistent; however, there is still some misalignment in the acoustic signals and a maximum error of around 0.25 m. The limitations of the *features* method are shown by the estimation of the robot’s velocity. As the method adds no information between the features, the optimization must use only a fixed mean velocity, which is seen to differ from the real drifting velocity.

The optimized trajectory shown in Figure 6b uses a combination of the phase-correlation and quadratic fit methods. It is shown to be mostly consistent in the two directions along the pipe and is close to the true value. This is illustrated by the matching estimate of spatial signal, by low variance of error, and by the good match between the noisy velocity and the estimated velocity. Compared to the *features* estimate, the variance in error is much lower as the trajectory estimate is very consistent and the error in the estimate is generally lower.

**Figure 7:**
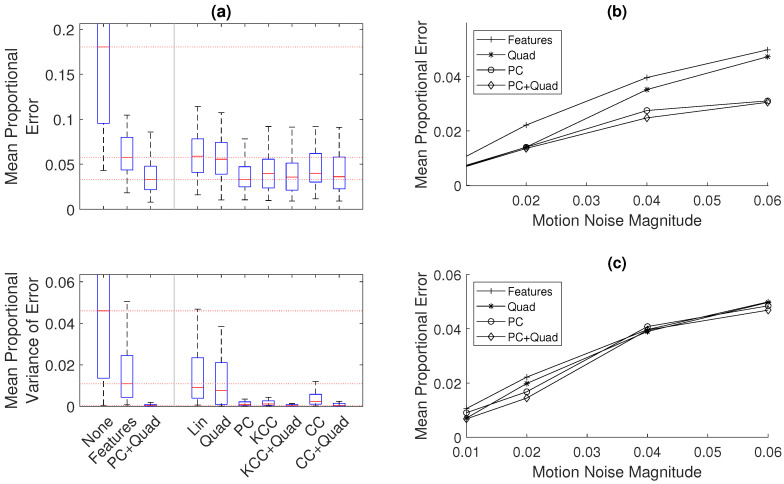
A comparison of methods used to improve the trajectory estimate in a single 1D pipe: in all cases, *proportional* measures of error are used, which are normalized by the length of the pipe, to more easily allow comparison to other results. (**a**) A comparison of the benchmark pipe-feature method with the different developed spatial field information methods in the 5-m pipe for a motion noise magnitude of 0.06 of the command motion, using box plots to show the quartiles of results over 50 sets of random noise: the top graph shows the error in the estimation, and the bottom graph shows the inconsistency in the estimation. On the left is the unoptimized (*None*), benchmark (*Features*), and improved estimate (*PC+Quad*) results. On the right is the results found using the other methods presented in this paper. (**b**,**c**) The median accuracy for each of the methods for the 5- and 20-m pipes respectively, over a range of noise magnitudes: the variance of the normally distributed noise added to the motion at each time step is equal to the noise magnitude multiplied by the command motion.

The main result is shown in Figure 7, where the methods are compared using the same fifty sets of random noise. Figure 7a shows that the median error is 74% lower when using only the pipe ends as *features* compared to the simple estimate using no optimization. The use of additional information methods (*quadratic fit* and *phase-correlation* (*PC+Quad*)) is shown to give a median error that is 84% lower than the error without optimization. The method presented here therefore has a median error 39% lower compared to the typical optimization method using only feature measurements.

In this case, it is seen that the use of the *linear fit* and *quadratic fit* methods alone are shown to give only a small increase in accuracy. The use of *cross-correlation* (*CC*) and *kernel cross-correlation* (*KCC*) gives a larger improvement to accuracy, and the use of *phase-correlation* (*PC*) and the combination of the *quadratic fit* and both *cross-correlation* (*CC+Quad*) and *kernel cross-correlation* (*KCC+Quad*) give a large improvement to accuracy, similar to that of the *PC+Quad* method. These results for error, the overall aim of the pose-graph optimization, are reflected in the measure of variance of error or in consistency in the acoustic measurements. The use of the *PC+Quad*, *CC+Quad*, and *KCC+Quad* methods are shown to give a very consistent estimate.

Figure 7b,c expand this result to different levels of motion noise. They show a comparison between this *features*-only method and the methods using *phase-correlation* and *quadratic fit*. In Figure 7b, which shows the comparison for a 5-m pipe, it is seen that the use of the *PC+Quad* and *PC* methods gives a consistent improvement to accuracy across a range of noise, while the accuracy when using the *Quad* method decreases towards the benchmark accuracy with increasing motion noise.

Figure 7c shows the shows the comparison for a 20-m pipe. As in the 5-m case, as the noise increases, the performance of the *Quad* method decreases more than that of the other methods, but it is seen that the accuracy of all methods decreases to that of the benchmark *features*-only method for large values of motion noise. For the lower magnitudes of motion noise, the combination of the *PC+Quad* method gives a lower error compared to using only the *PC* method.

An increase in motion noise increases the initial misalignment of the signal measured in each direction along the pipe and an increase in the warping of the signal. As expected, the use of *phase-correlation* is required for improving the initial alignment of the signal so that further improvement can be found using the *quadratic fit*. However, it is seen that, at large values of noise for the fairly periodic 20-m signal, the signal in each direction is warped enough so that it is difficult for the method to find matching sequences of measurements, and therefore, the accuracy is not improved.

### 4.2. Sensitivity to Measurement Noise

**Figure 8:**
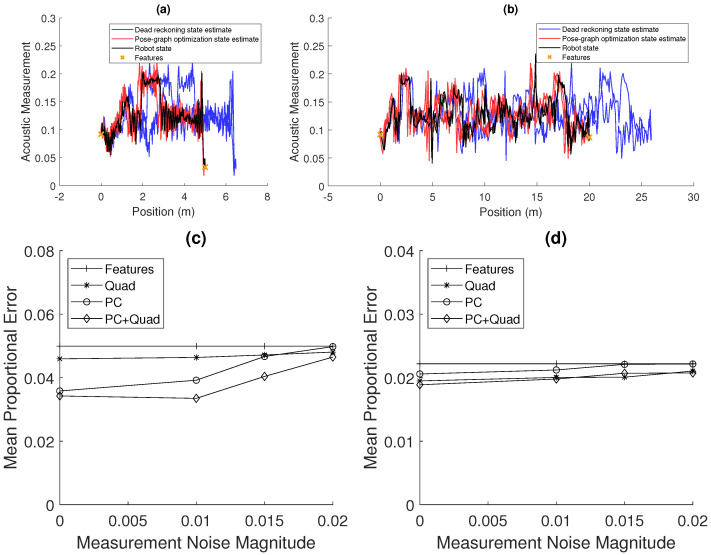
Results showing the robustness of the developed methods to noise in the measurement of spatially varying acoustic property: (**a**,**b**) example recorded acoustic signals over 5 m and 20 m with additive normally distributed noise with a standard deviation of 10 percent of the signal amplitude. (**c**,**d**) An analysis of the sensitivity of the estimation method to noise in the acoustic signal: for varying measurement noise magnitude (the variance of the normally distributed noise added to the signal measurement), a measure of the mean error was measured for both 5 m (with 0.06 motion noise) (**c**) and 20 m (with 0.02 motion noise) (**d**) pipes. The median is shown for each method. Slightly different parameters are used for the *phase-correlation* method compared to the results in Figure 7, reducing the threshold to which matches are compared, which allows matches to be found in the presence of noise.

Figure 8 shows an analysis of the sensitivity of the estimation to noise in the acoustic signal. Figure 8a,b shows examples of the acoustic signal corrupted with noise. Figure 8c,d shows how the performance of the *Quad*, *PC*, and *PC+Quad* methods varies with measurement noise magnitude. These are compared to the results using the *features*-only method, which should be unchanged with variation in measurement noise.

Figure 8c shows that, for the 5-m case with a motion noise of 0.06, the performance of all methods decreases with increasing measurement noise. However, the performance of the *PC+Quad* method decreases slower than the performance of the *PC* method, which shows that the former method is more robust to measurement noise. Figure 8d shows similarly that, for the 20-m case with a motion noise of 0.02, the performance of all methods decreases. However, the performance found that using the *Quad* method and *PC+Quad* method does not degrade to that of the *features*-only method. These results show the advantage of the addition of the *quadratic fit* method. It gives a robustness to noise unlike the *phase-correlation*-based method, which struggles to match sequences of measurements when corrupted by noise.

## 5. Discussion

### 5.1. Summary of Results

This work has developed a pose-graph optimization algorithm that incorporates an acoustic signal for pipe robot localization. The pose-graph optimization cost function in Equation (9) was augmented with a novel term for incorporating information from a measured acoustic property that varies along the robot’s trajectory. Four specific implementations of this novel term were developed and are compared in the results (linear fit, quadratic fit, phase-correlation, and cross-correlation). It was found that the combination of a quadratic fit *prediction* term and a phase-correlation *matching* term gives the best results, reducing the average error by 39% compared to a typical pose-graph optimization formulation without the acoustic signal. This is equivalent to a reduction in error of 84% compared to dead reckoning, where typical pose-graph optimization gives a reduction in error of 74%.

The phase-correlation *matching* method is seen to be more effective at higher magnitudes of motion noise, which is expected as the initially poorly aligned signal estimates would benefit more from approximate global alignment. The *prediction* method, especially in combination with the *matching* method, is seen to be more effective at larger magnitudes of measurement noise where the *matching* methods are less able to find matching sequences of measurements.

### 5.2. Comparison of Results

This quantitative result is difficult to compare to results found in the literature. The most comparable results are from previous work using an acoustic signal to build a map for localization in a pipe [26] and other work using a magnetic field to improve localization in a drilling robot [14]. In both cases, as in this work, the robot travels twice alone the same path and uses a continuous spatially varying field to improve localization. However, there are differences in the aims and measures used in these other works that make a useful quantitative comparison difficult to make.

Where this work achieves an 84% reduction in error compared to dead reckoning, in the previous work using an acoustic field in a pipe, the error is reduced by 78%. However, in this previous work, the aim is for *online* localization rather than *full* localization. A measure of the total error would be larger for an *online* method than a *full* method; therefore, a comparison between these values is not conclusive.

In the case of the drilling robot, the error is reported to be reduced by 81% and 98% in the two experiments. However, in this case, the robot’s position is estimated in three dimensions rather than one, so the dead reckoning error is very large as the uncertainty in motion in each of the dimensions adds to the error when measured as Euclidean distance. Therefore, pose-graph optimization should give a much larger relative improvement in a larger number of dimensions, so a comparison between the values for relative reduction in error is not conclusive between three dimensions and one dimension. Conceptually, however, the method presented in this paper offers a useful addition to this previous work, as it uses a similar correlation *matching* method in combination with additional pose-graph information using the quadratic fit *prediction* method.

### 5.3. Challenges and Implications

This section will describe the challenges found in implementation of the proposed methods, and the implications in relation to the wider literature will be discussed.

#### 5.3.1. Experimental Challenges

An acoustic signal for robot localisation in water pipes was used in our previous work, using an Extended Kalman Filter [25] and a Rao-Blackwellised Particle Filter [26]. However, those studies used a short length of pipe, 0.5 m, whilst this study used a 5-m length of pipe. The results from this longer section of pipe have confirmed and extended the results from those studies, namely that the use of the acoustic signal improves substantially on the use of dead reckoning alone. There is a limitation in the experimental setup, which is a relatively short section of straight pipe. Ideally, a network of pipes should be used in the future to test the localisation method, including bends and junctions. However, it is technically challenging to demonstrate robot localisation in water-filled pipes and, therefore, few published works have done so.

#### 5.3.2. Challenges in Constructing the Optimization Problem

The *true* aim of robot localization is to obtain an accurate trajectory estimate. The methods presented here aim to estimate a trajectory such that the acoustic property measurements are consistent, which should reduce actual errors in the estimate. This is much like the typical pose-graph optimization problem in other applications, where the trajectory estimated is consistent with respect to observations of features, which should reduce the actual error in the estimate given the motion and measurement models.

In pose-graph optimization, the *implied* optimization problem is to find an accurate, consistent trajectory estimate. With the addition of the *information* methods presented here, this is not implemented directly and the *constructed* optimization problem is to find a trajectory estimate which gives a consistent estimate of the spatially varying acoustic signal given the specific costs associated with the *information* terms.

**Figure 9:**
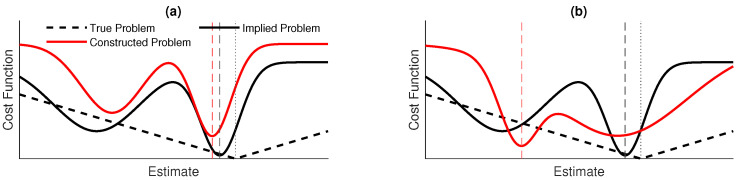
A simplified illustration of the cost functions considered in this work: in this example, the aim would be to find the estimate which minimizes the cost function. The quality of a cost function is measured by how close its minimum is to the minimum of the *true* problem, which would correspond to the correct estimate. However, this is not possible directly. The *implied* problem is to find a trajectory estimate which is consistent with respect to measurements. This is not done directly and, instead, a cost function is defined, creating the *constructed* problem using the functions defined in Section 3.3. Shown are (**a**) A *constructed* problem which matches well with the *implied problem* and (**b**) a bad *constructed* problem.

The aim of this work therefore has been to find specific functions for the *constructed* optimization problem such that it finds a good solution to the *implied* optimization problem without making any general assumptions about the measurement and motion models. This is illustrated in Figure 9.

#### 5.3.3. Challenges from Additional Dynamics

**Figure 10:**
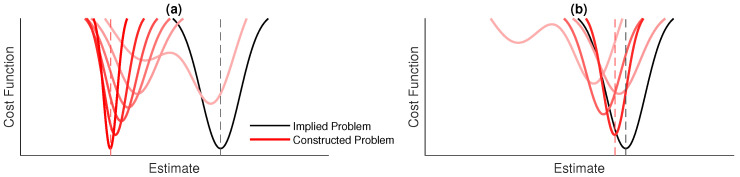
A simplified illustration of the dynamic cost functions considered in this work: the *constructed* problem is designed to be a good match to the *implied problem*, so that the minimum of the *implied* problem can be approximately found. However, the *constructed* cost function is dynamic and changes as the optimization progresses. Illustrated are (**a**) the cost function diverging from the *implied* function and (**b**) the cost function converging towards a good approximation to the *implied* function.

The dynamics from the information method functions can cause two types of divergence that make optimization more difficult: firstly, divergence *of* the solution of the *constructed* problem from the solution of the *implied* problem, which is illustrated in Figure 10, and secondly, divergence *from* the solution of the *constructed* problem.

The typical pose-graph optimization problem (described by Equation (6)) is a nonlinear least squares regression problem where the static data points are the noisy measurements and the model to be fit to the data is the set of robot poses. The least squares problem is iteratively solved until the solution converges. The addition of the information methods (described by Equation (9)) gives some dynamic behaviour. The data points from the *matching* and *prediction* functions are dependent on the model being fit to the data and, therefore, change at each iteration of the solution.

Poor performance of the information methods can lead to divergence of the solution from the implied optimal estimate. For example, an incorrect match between two poses could lead to warping of the estimated spatial signal and error in subsequent matching. In the least squares regression sense, this is an update to the model which causes an increase in the residuals due to the consequent update of some of the data points.

The divergences can be controlled by the optimization algorithm. The methods here use the *GraphSLAM* algorithm [36], which is analogous to the Gauss–Newton method, which is locally convergent for mildly nonlinear problems [39] but divergent for problems that are highly nonlinear or have large residuals. The damped Gauss–Newton method [39] reduces the magnitude of the model update at each iteration with a parameter $\alpha_{GN}$. Similarly, the Levenberg–Marquardt method [38] uses a parameter $\lambda_{LM}$ to vary the magnitude and direction of the model update.

In this work, rather than using a variable parameter, the damping is based on information used at previous iterations of the optimization. Instead of re-initializing the information form parameters Ω and ξ at each iteration, the previous information is kept and new information is added. Information that is found repeatedly is reinforced as the optimization progresses, while the repeated addition of the static information from motion and landmark measurements causes the dynamic optimization to become more rigid. The resulting method has similarities to using a decaying value for $\alpha_{GN}$ or $\lambda_{LM}$ in the above methods.

Similar dynamics would be found in other formulations of pose-graph optimization, and this solution is more generally applicable. For example, where the current estimated distance between two points is a factor in determining a loop closure, the cost function is changed during the optimization process.

#### 5.3.4. Challenges from Sensitivity to Parameters

As in any other localization method, the proposed methods have some sensitivities to parameters, some of which are possible to address.

The *matching* methods are sensitive to the choice of window size, and it is difficult to address by using a range of window sizes as smaller windows are likely to produce a larger number of false matches which need to be accounted for. The *matching* methods are also sensitive to the means of taking a measurement between robot poses from the matching subsets of the spatial signal. The measurement is made between poses at the centres of the matching subsets, although the information used to find the match is distributed across the whole subset and other pose pairs might be better matches. The *matching* methods require a choice of parameters for interpolation of the spatial signal and for the number of points used in each subset. The *matching* process could also be achieved using methods other than correlation, such as a Euclidean distance measure between two sequences as used in other work with magnetic fields [33,40].

The *prediction* methods are also sensitive to the window size to which they are applied. However, it is simple to simultaneously use a range of window sizes. The *prediction* methods are also sensitive to the choice of function fit to the data. Any differentiable function could be implemented; however, in this work a quadratic fit has been chosen as a lower order (linear) fit which might exhibit underfitting and a higher order polynomial fit which might exhibit overfitting. If more was known about the spatially varying signal, then a more informed choice of function might be made. A Gaussian process, as used in another work on a excavating robot [40], or a weighted radial basis function decomposition, as used in a previous work in pipes [26], may be more flexible functions.

### 5.4. Integration with Additional Information

#### 5.4.1. Integration with Additional Sensors

The use of pose-graph optimization would allow easy integration of other sensor information. Information could simply be added to the cost function in Equation (9) if the state vector x is extended to include more features in the environment as necessary.

Pose-graph optimization has been applied to localization of robots in pipes using vision [7], a fusion of IMU and tether cable information [17], and periodic radio wave amplitude [41]. Recent work in the use of acoustic echoes for localization in pipes [42] could be applied to pose-graph optimization; these acoustic measurements are range-only measurements of features, which have been used in pose-graph optimization in other applications [43,44]. As applied in many of these methods, the use of an approach based on the Levenberg–Marquardt method, such as *general graph optimization* [45], may be sufficient to find an optimal trajectory estimate given the dynamic information presented in this paper.

#### 5.4.2. Adaptation to Two or Three Dimensions

The methods presented here have been developed as an augmentation of the typical pose-graph optimization problem so that extension to two or three dimensions and the addition of prior knowledge can be done without significant further alteration of the method, while knowledge of the position of the whole pipe would reduce the problem to one dimension. Other methods of incorporating prior knowledge of the position of the pipe have been demonstrated in a recent work on localization of robots in sewer systems [46] and in a work on localization of autonomous road vehicles [47,48,49,50].

## 6. Conclusions

The use of pose-graph optimization for localization of a robot in an underground water pipe has been demonstrated. As an alternative to visual localization methods, four methods of incorporating information from the measurement of an acoustic spatial field are presented, described, and compared using simulations based on experimental acoustic measurements. The developed methods are designed to be applicable to any spatially varying property along the robot’s trajectory, such as magnetic or electric fields.

The proposed best method for incorporating this acoustic information is a combination of a correlation-based matching method and a quadratic fit-based prediction method. Compared to the standard pose-graph optimization method using typical features which gives an 74% lower average localization error compared to dead reckoning, the presented methods of incorporating spatial field measurements have an 84% lower average localization error. The use of matching-based methods is seen to give a larger improvement to accuracy at higher magnitudes of motion noise, while the use of prediction-based methods gives an improved robustness to measurement noise.

The study presented here has demonstrated the advantages of using the acoustic signal with pose-graph optimization; however, there were challenges to overcome in the implementation of this method. The first challenge was that the addition of the information methods in Equation (9) can affect the convergence of the estimation algorithm: the data points from the *matching* and *prediction* functions are dependent on the model being fit to the data and, therefore, are dynamic and change at each iteration of the solution. This can lead to an increase in the residuals and divergence of the estimation algorithm, and therefore, more robust optimization algorithms are considered. The second challenge was the optimal choice of parameters for the information methods, such as the size of sequences of measurements to be matched or the choice of function to fit to the acoustic signal. Simultaneously using a range of parameters reduces the sensitivity to the choice of parameters at the cost of computational complexity.

Further work on this topic could be done. The use of pose-graph optimization in this work allows easy integration of other sensor measurements, so improvements to the results could be found by integrating visual and inertial sensing. The experimental evaluation could be improved by further real-world testing in a number of larger scale pipes to investigate the robustness of the method to a range of acoustic data. The method could also be extended to acoustic sensing in plastic pipes, where the hydrophone-induced vibration found in metal pipes could be replaced with ultrasonic sensing which can penetrate the plastic pipe. The sensitivity of the method to the parameters described could be investigated, and a means of finding an optimal set of parameters could be developed.
